# Postoperative nausea and vomiting and recovery of heart rate variability following general anesthesia with propofol or sevoflurane: a randomized, double-blind preliminary study

**DOI:** 10.3389/fmed.2025.1575865

**Published:** 2025-04-30

**Authors:** Hyoeun Ahn, Yun Jeong Chae, Seyoon Kang, In Kyong Yi

**Affiliations:** Department of Anesthesiology and Pain Medicine, Ajou University School of Medicine, Suwon, Republic of Korea

**Keywords:** anesthesia, autonomic nervous system, inhalational anesthesia, postoperative nausea and vomiting, propofol

## Abstract

**Purpose:**

Postoperative nausea and vomiting (PONV) remain significant complication after general anesthesia. Inhaled anesthetics are a known risk factor for PONV. Heart rate variability (HRV), an indicator of autonomic nervous system balance, is influenced by anesthetic agents and may be linked to PONV. This study aimed to compare HRV dynamics and their relationship with PONV between sevoflurane and propofol anesthesia.

**Methods:**

This preliminary randomized double-blind study included 40 adult participants aged 20–60 years, with the American Society of Anesthesiologist Physical Status I or II, scheduled for elective breast surgery; the participants were randomly assigned to sevoflurane or propofol group. HRV was measured preoperatively and postoperatively, in the post-anesthetic care unit. Incidence (defined as the presence or absence of any PONV during the study period), severity (0–3 points), PONV impact scale, and patient satisfaction, were recorded postoperatively at 30 min, 3 h, and 1 and 2 days.

**Results:**

There was no significant intergroup difference in demographics, type of surgery, anesthesia duration, and Apfel score. The sevoflurane group had a higher PONV incidence than the propofol group; significant intergroup differences in the PONV profile were mainly observed <30 min postoperatively in PONV frequency and severity and nausea severity. Although there was no significant intergroup difference in preoperative and postoperative frequency-domain parameters, total power (Ln), LF, LF (Ln), and HF (Ln), decreased postoperatively in the sevoflurane group.

**Conclusion:**

Sevoflurane use was associated with more frequent, severe PONV <30 min postoperatively than propofol use, without differences in HRV dynamics. With sevoflurane use, HRV parameters did not recover to preoperative levels and were potentially associated with PONV.

## 1 Introduction

Postoperative nausea and vomiting (PONV) constitute an unfavorable component of the perioperative experience (1). PONV affects patient satisfaction and can prolong hospital stay, lead to unplanned hospitalization, and increase overall costs. Various neurotransmitter pathways, including serotonin 5-HT3, dopamine D2, and histamine H1, mediate the mechanisms underlying PONV. As the neuronal pathway that will be involved is unpredictable, a multimodal strategy that targets diverse mechanisms is recommended, especially in high-risk patients (2). Risk factors for PONV include female sex, history of PONV, nonsmoking, type of surgery (e.g., laparoscopic, gynecologic), opioids, and inhaled anesthetics (2). Inhaled anesthetics, such as sevoflurane, may cause PONV in the first 2 h postoperatively (3). Although the pathomechanism of inhalational anesthesia-induced emesis or nausea remains unclear, theories of the pathogenesis have implicated stimulation of vagal afferent fibers, enhanced 5-HT3 receptor function, or vestibular changes (4–6). Propofol, an intravenous anesthetic that is used as frequently as an inhaled anesthetic, has antiemetic properties, and replacing an inhalational agent with propofol reduced the incidence of PONV (7, 8).

The autonomic nervous system (ANS) mediates nausea and vomiting (9–11). Patients with vagotomy who underwent surgeries developed less PONV, which suggested the possibility that vagus nerve-dependent gut–brain signaling mainly contributes to PONV (11). Moreover, increased parasympathetic activity seemed to be related to cancer chemotherapy-associated nausea (10). Heart rate variability (HRV) is a sensitive, noninvasive marker of autonomic nervous system (ANS) activity, reflecting beat-to-beat variations in heart rate modulated by multiple regulatory mechanisms. The high-frequency (HF) component indicates parasympathetic activity, while the low-frequency (LF) component reflects both sympathetic and parasympathetic influences. Total power represents overall autonomic activity, and the LF/HF ratio is often used as an index of sympathovagal balance, though it requires cautious interpretation due to the complex nature of autonomic regulation. With its ability to provide real-time insights into ANS function, HRV is a practical and effective tool for this study (12). Based on the anesthetic agent that is used, HRV responds differently (13); although propofol reduces cardiac parasympathetic activity, depending on the depth of anesthesia, sevoflurane has little or no effect on the cardiac parasympathetic tone (13). Although both propofol and sevoflurane influence autonomic tone during anesthesia, the differential effects of these agents on HRV in the recovery phase—and their potential association with PONV—remain poorly understood. To our knowledge, no previous study has directly compared the postoperative HRV dynamics between sevoflurane and propofol anesthesia in relation to PONV.

We designed this study with the hypothesis that understanding the differences in ANS dynamics according to anesthetic agents, and their relationship with PONV, may open the possibility of adopting ANS modulation as a novel approach for the prevention or management of PONV. Specifically, we hypothesized that the lower incidence of PONV with propofol compared to sevoflurane is associated with differences in ANS status during recovery. Therefore, this preliminary study aimed to investigate HRV dynamics according to anesthetic choice and to clarify the relationship between HRV and PONV.

## 2 Materials and methods

The study was approved by the Ethics Committee of Ajou University Hospital (AJOUIRB-OBS-2020-293, 3 August 2020) and was registered at clinicaltiral.gov (NCT04514211, 12 August 2020). Adult patients aged 20–60 years, with the American Society of Anesthesiologist (ASA) physical status class I or II, who were scheduled for elective breast surgery under general anesthesia were enrolled and provided written informed consents for study participation. Patients with arrhythmia, diabetes, hypertension, and thyroid dysfunction were excluded as were those who were taking medications, such as psychiatric medications (e.g., antidepressants, antipsychotics, and anxiolytics) or beta blockers, that could affect HRV, and were unable to cooperate with the 3 min HRV measurement.

A total of 40 participants were recruited and randomly assigned to either the propofol or sevoflurane group using a random number table. Allocation concealment ensured by sealed, opaque envelopes prepared in advance by an independent researcher who was not involved in participant recruitment or data analysis. Heart rate variability (HRV) was assessed by one of the authors (SK), and postoperative nausea and vomiting (PONV) was evaluated by one of the authors (YC), both of whom were blinded to group allocation. For patients who were discharged early, telephone follow-up was conducted postoperatively by the same blinded investigator (YC), using a standardized questionnaire to assess PONV. Patients were blinded to their group allocation, and only the attending anesthesiologist (IY or HA) who administered the general anesthesia was aware of the assignment. All outcome assessors remained blinded to group allocation throughout the study.

Demographic characteristics, including age, sex, body mass index (BMI), Apfel PONV score, and anesthesia duration, were collected and compared between the two groups.

### 2.1 HRV measurement

As per the institutional protocol, patients who visited the hospital on the morning of the surgery provided written informed consent upon arrival at the daycare surgery center. To minimize confounding bias owing to the circadian rhythm, we only enrolled patients who underwent surgery before noon. After their transfer to the surgery-preparation room and completion of the patient-identification procedure, HRV was measured in a quiet isolated room for 3 min using the SA-3000P (Medicore Co., Ltd., Hanam, Gyeonggi-Do, Republic of Korea), a clinically validated device that applies a Fast Fourier Transform (FFT) algorithm for frequency-domain analysis. Patients rested in the supine position for 5 min prior to measurement and were instructed to close their eyes, breathe comfortably, and avoid speaking or moving during the recording. Although often categorized as an ultra-short-term measurement, 3-min HRV analysis has been shown in previous studies to provide reliable and representative parameters (14, 15). This duration was selected as it balanced clinical feasibility with analytical validity.

The second HRV measurement was performed 30–40 min after PACU arrival, in accordance with our institution’s average post-anesthesia care unit (PACU) stay of around 40 min. This time frame was selected because patients are often not fully cooperative immediately after PACU arrival, which could result in unreliable HRV data. The same HRV parameters were measured preoperatively and postoperatively, and automatically calculated using SA-3000P. The time-domain parameters were mean interval, standard deviation of the NN interval (SDNN), and root mean square of the successive differences (rMSSD) whereas the frequency-domain parameters were total power, low-frequency power (LF; 0.04–0.15 Hz), high-frequency power (HF; 0.15–0.4 Hz), LF/HF ratio, and Ln (a natural log).

### 2.2 Anesthetic and postoperative management

Upon entering the operating room, the patient underwent noninvasive monitoring, including electrocardiographic, blood pressure, pulse oximetry, and bispectral index (BIS, Medtronic, Minneapolis, MN, USA) measurements. After preoxygenation with 6 L/min oxygen, anesthesia was induced. Propofol and remifentanil were administered using a target-controlled infusion (TCI) system, employing the Schnider pharmacokinetic model for propofol and the Minto model for remifentanil. In the propofol group, using a TCI to achieve a target effect-site concentration of 4 ug/mL propofol. and 3 ng/mL remifentanil. After loss of consciousness, with ventilation, rocuronium (0.6 mg/kg) was administered, and 90 s later, endotracheal intubation was performed. Anesthesia was maintained using continuous TCI with propofol (2–5 μg/mL) and remifentanil (2–4 ng/mL), titrated to maintain a BIS between 40 and 60. In the sevoflurane group, anesthesia was induced by thiopental (4 mg/kg) and remifentanil (3 ng/mL, TCI using the Minto model). After loss of consciousness, rocuronium (0.6 mg/kg) was administered, and the patient was ventilated with sevoflurane (end-tidal concentration 4%) in oxygen and, after 90 s, endotracheal intubation was performed. The sevoflurane concentration was adjusted to maintain the BIS level between 40 and 60 (end-tidal MAC of 0.8–1.2), and remifentanil was titrated to 0–2 ng/mL to keep HR and BP within 20% of the baseline values. While the total intraoperative dose of remifentanil was not recorded, its ultra-short-acting pharmacokinetic profile reduces the likelihood that differences in dosing between groups significantly affected the primary outcome of PONV incidence. The intraoperative administration timing and dose of vasoactive drugs were recorded.

After completion of surgery, patients in both groups were administered sugammadex 2 mg/kg for neuromuscular blockade reversal and were extubated once adequate spontaneous breathing was confirmed. They were then transferred to the PACU for postoperative monitoring.

Postoperative rescue protocols in the PACU were as follows: If a patient reported a verbal rating scale (VRS) pain score ≥5, Ketorolac 30 mg IV was administered. If the pain remained ≥5 after 15 min, an additional fentanyl 50 μg IV was given. For nausea and vomiting, if the VRS for PONV was ≥5, metoclopramide 5 mg IV was administered as a rescue antiemetic.

Discharge from the PACU was based on achieving an Aldrete score ≥9, in accordance with institutional practice.

In the general ward, patients received standard postoperative care according to the routine protocol of the breast surgery department. This included ramosetron 0.3 mg IV immediately upon arrival to the ward and ketorolac 30 mg IV every 8 h on postoperative day 1.

### 2.3 PONV measurement

Participants responded to questionnaires on PONV after 30 min, 3 h, 1 day, and 2 days after surgery. The questionnaires were administered through interviews where participants were asked to self-rate their scores. If the patient was discharged within 2 days, the PONV questionnaire completion was conducted telephonically. Moreover, the overall satisfaction regarding PONV was investigated 2 days after surgery.

The incidence of PONV was defined as whether or not any episode of nausea or vomiting occurred during the study period.

Using PONV Impact scale (16), Frequency of nausea and vomiting were graded on a 3-point scale as follows: 0 = none, 1 = once, 2 = twice, and 3 = ≥ 3 times. When measuring the number of occurrences, multiple symptoms that occurred within 5 min were measured as a single event. In addition, the degree of nausea and vomiting was graded on a 3-point scale as follows: 0 = none, 1 = sometimes affected daily life, 2 = frequently affected daily life, and 3 = continuously affected daily life. Patient satisfaction regarding PONV was evaluated using the Numeric Rating Scale (NRS) on a scale of 0 to 10: 0 = very dissatisfied; 10 = very satisfied.

In accordance with the multimodal PONV preventive strategy, all patients received 0.3 mg ramosetron intravenously upon arrival at the ward after discharge from the PACU. On the first postoperative day, all patients received 30 mg ketorolac intravenously to preclude opioid administration.

### 2.4 Statistical analysis

Statistical analysis was undertaken using R software (version 4.0.5; R Foundation for Statistical Computing, Vienna, Austria). Demographic variables were expressed as mean ± standard deviation (SD), and comparisons between groups were performed using the independent *t*-test. For heart rate variability (HRV) parameters and clinical outcomes, non-parametric statistical methods were employed due to non-normal distribution. The Wilcoxon signed-rank test was used for within-group comparisons, and the Mann–Whitney U test was applied for between-group comparisons. A Bonferroni correction was applied to adjust for multiple comparisons.

## 3 Results

### 3.1 Demographics

**FIGURE 1:**
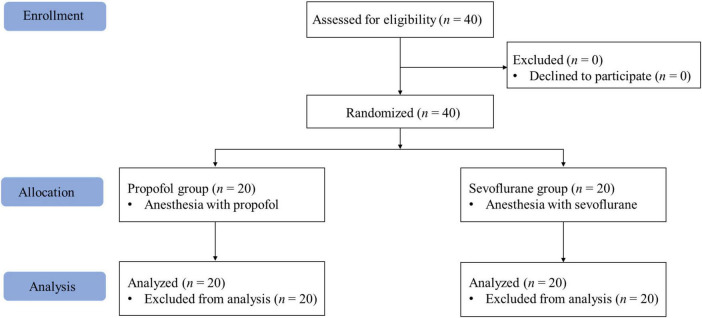
Flow diagram.

All 40 patients who were randomized to the study groups completed this study (Figure 1). There were no significant intergroup differences in demographic data, type of surgery, duration of anesthesia, and Apfel score (Table 1). Among the patients, two cases of radical mastectomy with axillary lymph node dissection were performed, one in the sevoflurane group and one in the propofol group. Vasoactive drug use was comparable between the two groups. Ephedrine was the primary agent administered, and there were no significant differences in either the incidence of use or the total dose between the groups.

**TABLE 1:** Characteristics of the study participants.

Characteristic	Propofol (*n* = 20)	Sevoflurane (*n* = 20)	*P*-value
Age, years	49.8 ± 4.6	47.2 ± 7.5	0.197
Height, cm	159.0 ± 6.0	156.9 ± 5.3	0.309
Weight, kg	52.8 ± 5.2	55.4 ± 9.4	0.464
Surgery type, *n* (%)			0.737
Mastectomy	0 (0)	2 (10)	
Partial mastectomy	19 (95)	17 (85)	
Radical mastectomy	1 (5)	1 (5)	
Anesthesia duration, min	95 [80–109]	95 [81–105]	0.793
Apfel PONV Score, *n* (%)			0.856
2	13 (65)	13 (65)	
3	3 (15)	2 (10)	
4	4 (20)	5 (25)	
Fentanyl use in PACU, *n* (%)	5 (25)	5 (25)	>0.999

Data are presented as the mean ± standard deviation, frequency (proportion), and median [interquartile range]. PONV, postoperative nausea and vomiting. Apfel PONV score (0–4): based on female sex, history of PONV/motion sickness, nonsmoking, and postoperative opioid use. Fentanyl use in PACU, refers to the incidence of patients who received intravenous fentanyl for pain management during their stay in the postanesthesia care unit (PACU).

### 3.2 Propofol vs. sevoflurane

There was no significant intergroup difference in preoperative and postoperative hemodynamic data or HR and time-domain parameters. Within each group, there was no significant difference between the preoperative and postoperative data parameters. In the sevoflurane group, however, the postoperative DBP was higher than the preoperative DBP (71 [65–76] mmHg vs. 77 [73–80] mmHg, adjusted *p* = 0.021; Table 2). The PONV profile is presented in Table 3. The incidence of PONV was 10.0% (95% CI, 1.2–31.7%) in the propofol group and 35.0% (95% CI, 15.4–59.2%) in the sevoflurane group (*P* = 0.127). The intergroup difference in PONV profile was mainly evident <30 min after surgery. Within the first 30 min after surgery, both the frequency and severity of PONV, and the PONV impact Scale, were significantly higher in the sevoflurane group compared to the propofol group (frequency: 0 [0–0] vs. 0 [0–1.25], *p* = 0.043; severity: 0 [0–0] vs. 0 [0–1], *p* = 0.040; PONV impact scale: 0 [0–0] vs. 0 [0–2.25], *p* = 0.043). No significant differences were observed between groups at later time points.

**TABLE 2:** Hemodynamic data and time domain parameters.

Variable	Propofol (*n* = 20)	Sevoflurane (*n* = 20)	*P*-value	*P*-adj*
**Systolic BP, mmHg**				
Preoperative	151 [139–164]	140 [131–162]	0.317	0.633
Postoperative	152 [134–158]	139 [130–150]	0.137	0.273
*P*-value	0.341	0.184		
*P*-adj*	0.682	0.368		
**Diastolic BP, mmHg**				
Preoperative	71 [66–75]	71 [65–76]	>0.999	>0.999
Postoperative	75 [67–81.5]	77 [73–80]	0.464	0.929
*P*-value	0.086	0.010		
*P*-adj*	0.171	0.021		
**Mean BP, mmHg**				
Preoperative	94 [89–107]	99 [87–105]	0.935	>0.999
Postoperative	100 [89–109]	97 [92–103]	0.694	>0.999
*P*-value	0.472	0.837		
*P*-adj*	0.944	>0.999		
**HR, bpm**				
Preoperative	69 [62–78]	66 [60–78]	0.946	>0.999
Postoperative	64 [57–68]	64 [58–68]	0.839	>0.999
*P*-value	0.126	0.045		
*P*-adj*	0.252	0.090		
**SDNN, ms**				
Preoperative	36 [30–47]	41 [29–60]	0.457	0.914
Postoperative	29 [23–63]	37 [27–46]	0.756	>0.999
*P*-value	0.751	0.323		
*P*-adj*	> 0.999	0.645		
**RMSSD, ms**				
Preoperative	29 [18–39]	23 [18–30]	0.579	>0.999
Postoperative	20 [15–32]	17 [13–28]	0.441	0.881
*P*-value	0.444	0.380		
*P*-adj*	0.888	0.761		

Data are presented as the median [interquartile range]. BP, blood pressure; HR, heart rate; SDNN, standard deviation of the RR interval; RMSSD, square root of the mean of the sum of the square of differences between adjacent RR intervals.

*Adjusted *p*-values using Bonferroni correction for multiple comparisons. Statistical significance considered at *p* < 0.05.

**TABLE 3:** Postoperative nausea and vomiting profile.

Variable	Propofol (*n* = 20)	Sevoflurane (*n* = 20)	*P*-value
Incidence of PONV, *n* (%)	2 (10)	7 (35)	0.127
Frequency of PONV			
Score 0/1/2/3, *n*			
0–30 min	19/0/1/0	14/1/4/1	0.043
0.5–3 h	20/0/0/0	17/3/0/0	0.080
3–24 h	19/1/0/0	18/2/0/0	0.573
1–2 days	20/0/0/0	20/0/0/0	1.000
Severity of PONV			
Score 0/1/2/3, *n*			
0–30 min	19/1/0/0	14/5/0/1	0.040
0.5–3 h	20/0/0/0	17/2/1/0	0.081
3–24 h	19/1/0/0	18/2/0/0	0.573
1–2 days	20/0/0/0	20/0/0/0	1.000
PONV impact scale*			
Score 0/1/2/3/4/5/6, *n*			
0–30 min	19/0/0/1/0/0/0	14/0/1/4/0/0/1	0.043
0.5–3 h	20/0/0/0/0/0/0	17/0/2/1/0/0/0	0.081
3–24 h	19/0/1/0/0/0/0	18/0/2/0/0/0/0	0.573
1–2 days	20/0/0/0/0/0	20/0/0/0/0/0/0	1.000
Patient satisfaction	9.5 [9–10]	9.5 [8–10]	0.362

Data are presented as the median [interquartile range]. PONV, postoperative nausea and vomiting. Frequency sore, 0 = none, 1 = once, 2 = twice, 3 = ≥ 3 times. Severity score, 0 = none, 1 = sometimes affected daily life, 2 = frequently affected daily life, 3 = continuously affected daily life. PONV impact scale = frequency score+severity score at each time point.

*Data are presented as the number of patients (*n*) at each score level (0/1/2/3). Patient satisfaction, the Numeric Rating Scale on a scale of 0 to 10: 0 = very dissatisfied; 10 = very satisfied.

**FIGURE 2:**
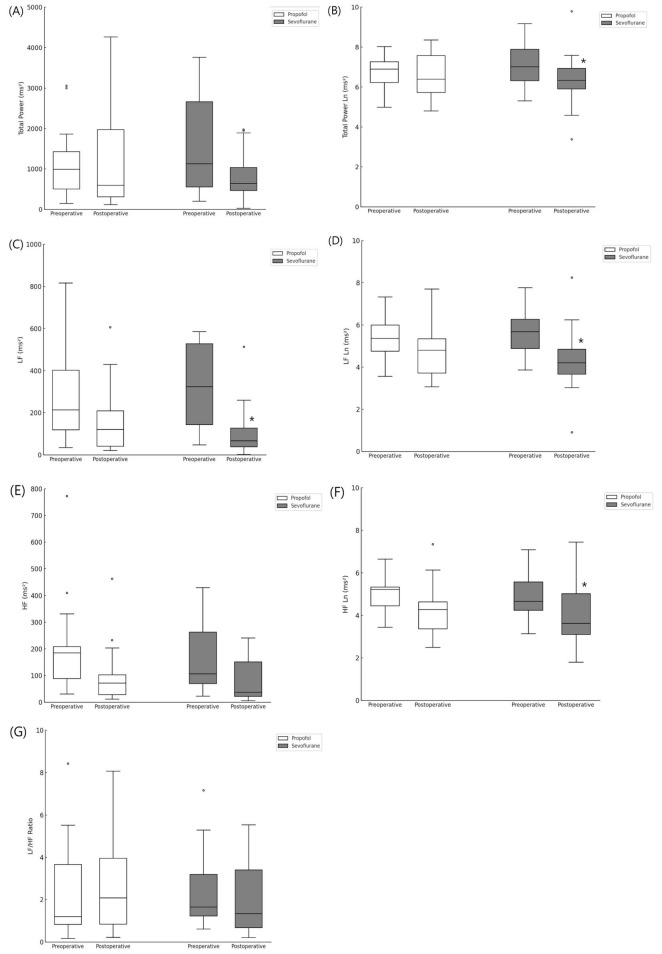
Frequency domain parameters. **(A)** Total power, **(B)** Total power Ln, **(C)** Low frequency, **(D)** Low frequency Ln, **(E)** High frequency, **(F)** High frequency Ln, **(G)** LF/HF ratio. *indicate a statistically significant difference compared with the preoperative value within the same group (Adjusted *p*-values using Bonferroni correction, *p* < 0.05).

The frequency-domain parameters are presented in Figure 2. There was no significant intergroup difference in preoperative and postoperative data. In the propofol group, there was no intragroup difference in preoperative and postoperative data. However, in the sevoflurane group, a significant intragroup difference was observed between the preoperative and postoperative data. Specifically, several HRV parameters decreased after surgery compared to preoperative values. Total power (Ln) decreased from 7.0 [6.3–7.9] ms^2^ to 6.3 [5.9–6.9] ms^2^ (adjusted *p* = 0.036). LF decreased from 325 [145–543] ms^2^ to 67 [36–133] ms^2^, and LF (Ln) from 5.7 [4.9–6.3] ms^2^ to 4.2 [3.66–4.85] ms^2^ (adjusted *p* = 0.002). HF (Ln) also decreased from 4.7 [4.3–5.6] ms^2^ to 3.6 [3.1–5.0] ms^2^ (adjusted *p* = 0.012). This indicates that some frequency-domain parameters show slower recovery to preoperative levels, which may be related to PONV. However, the LF/HF ratio showed no significant difference. The unchanged LF/HF ratio suggests that sympathovagal balance remained unaffected, despite overall reductions in autonomic tone.

### 3.3 No PONV vs. PONV

In total, 9 patients experienced PONV: 2 and 7 in the propofol and sevoflurane groups, respectively. There was no significant difference in patient characteristics between the PONV and no-PONV groups (Table 4). As expected, patient satisfaction was higher in the no-PONV group than in the PONV group (10 [9–10] vs. 8 [7–8], *p* < 0.001). The hemodynamic data and time-domain parameters are shown in Table 5, and revealed no significant intergroup difference between the no-PONV and PONV groups. On the intragroup analysis, diastolic BP increased postoperatively only in the PONV group (66 [61–76] vs. 77 [73–81] mmHg, adjusted *p* = 0.040). The mean HR decreased postoperatively in only the no-PONV group (68 [62–79] vs. 64 [58–67] mmHg, adjusted *p* = 0.034).

**TABLE 4:** Participant characteristics between patients with and without PONV.

Variable	No PONV (*n* = 31)	PONV (*n* = 9)	*P*-value
Anesthetics			0.127
Propofol, *n* (%)	18 (90)	2 (10)	
Sevoflurane, *n* (%)	13 (65)	7 (35)	
Age, years	49.3 ± 6.1	45.6 ± 6.4	0.236
Height, cm	158.2 ± 5.8	157 ± 5.7	0.649
Weight, kg	54.7 ± 11.8	56.6 ± 11.7	0.559
Surgery type, *n* (%)			0.091
Mastectomy	0 (0)	2 (22)	
Partial mastectomy	29 (94)	7 (78)	
Radical mastectomy	2 (6)	0 (0)	
Anesthesia duration, min	95 [80–105]	105 [90–125]	0.093
Apfel PONV score, *n* (%)			0.595
2	21 (67.7)	5 (55.6)	
3	3 (9.7)	2 (22.2)	
4	7 (22.6)	2 (22.2)	
Fentanyl use in PACU, *n* (%)	7 (23)	3 (33)	0.665
Patient satisfaction	10 [9–10]	8 [7–8]	<0.001

Data are presented as number (%), mean ± standard deviation. and the median [interquartile range]. PONV, postoperative nausea and vomiting. Apfel PONV score (0–4): based on female sex, history of PONV/motion sickness, nonsmoking, and postoperative opioid use. Fentanyl use in PACU, refers to the incidence of patients who received intravenous fentanyl for pain management during their stay in the postanesthesia care unit (PACU).

**TABLE 5:** Hemodynamic data and time domain parameters between patients with and without PONV.

Variable	No PONV (*n* = 31)	PONV (*n* = 9)	*P*-value	*P*-adj*
**Systolic BP, mmHg**				
Preoperative	151 [139–164]	131 [130–146]	0.070	0.140
Postoperative	142 [131–154]	149 [126–155]	0.859	>0.999
*P*-value	0.054	>0.999		
*P*-adj*	0.108	>0.999		
**Diastolic BP, mmHg**				
Preoperative	71 [67–76]	66 [61–76]	0.243	0.486
Postoperative	77 [69–81]	77 [73–81]	0.592	>0.999
*P*-value	0.029	0.020		
*P*-adj*	0.058	0.040		
**Mean BP, mmHg**				
Preoperative	96 [90–106]	85 [83–102]	0.092	0.184
Postoperative	97 [91–104]	77 [73–81]	0.820	>0.999
*P*-value	0.769	0.207		
*P*-adj*	>0.999	0.414		
**Mean HR, bpm**				
Preoperative	68 [62–79]	62 [60–76]	0.426	0.852
Postoperative	64 [58–67]	63 [58–73]	0.922	>0.999
*P*-value	0.017	0.398		
*P*-adj*	0.034	0.796		
**SDNN, ms**				
Preoperative	34 [29–55]	45 [35–51]	0.456	0.912
Postoperative	39 [25–56]	31 [24–37]	0.300	0.600
*P*-value	0.692	0.074		
*P*-adj*	>0.999	0.148		
**RMSSD, ms**				
Preoperative	23 [18–30]	31 [21–39]	0.257	0.514
Postoperative	22 [14–33]	18 [10–24]	0.136	0.272
*P*-value	0.915	0.027		
*P*-adj*	>0.999	0.054		

Data are presented as the median [interquartile range]. BP, blood pressure; HR, heart rate; SDNN, standard deviation of the RR interval; RMSSD, square root of the mean of the sum of the square of differences between adjacent RR intervals.

*Adjusted *p*-values using Bonferroni correction for multiple comparisons. Statistical significance considered at *p* < 0.05.

**FIGURE 3:**
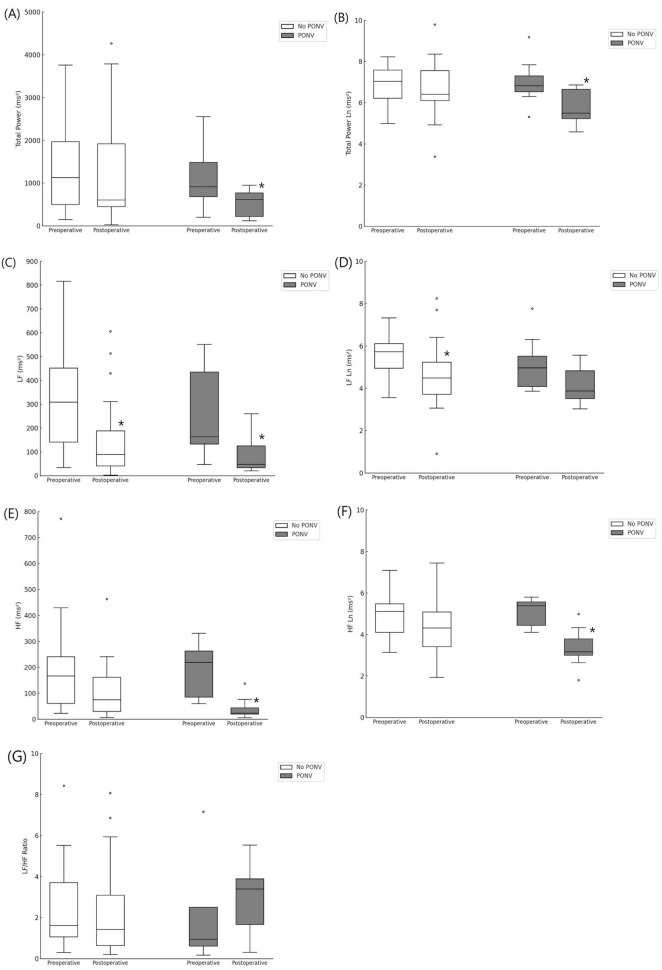
Frequency domain parameters between patients with and without PONV. **(A)** Total power, **(B)** Total power Ln, **(C)** Low frequency, **(D)** Low frequency Ln, **(E)** High frequency, **(F)** High frequency Ln, **(G)** LF/HF ratio. *indicate a statistically significant difference compared with the preoperative value within the same group (Adjusted *p*-values using Bonferroni correction, *p* < 0.05).

Figure 3 shows the frequency-domain parameters. There was no significant intergroup difference between the no-PONV and PONV groups. In the PONV group, total power, total power (Ln), LF, HF, and HF (Ln) all decreased after surgery, similar to the changes observed in the sevoflurane group. Specifically, total power decreased from 916 [615–2,021] ms^2^ to 615 [204–784] ms^2^ (adjusted *p* = 0.024). Total power (Ln) decreased from 6.8 [6.5–7.3] to 5.5 [5.2–6.7] ms^2^ (adjusted *p* = 0.008). LF decreased from 164 [97–493] to 48 [33–144] ms^2^ (adjusted *p* = 0.040), while HF decreased from 219 [75–263] to 24 [17–60] ms^2^ (adjusted *p* = 0.008). HF (Ln) also showed a significant decrease, from 5.4 [4.5–5.6] to 3.2 [3.0–3.8] ms^2^ (adjusted *p* = 0.008). In contrast, in the no-PONV group, only LF (Ln) showed a significant decrease, from 5.7 [5.0–6.1] to 4.5 [3.5–5.2] ms^2^ (adjusted *p* = 0.008). These results suggest that diminished recovery of frequency-domain HRV parameters may contribute to the development of PONV, irrespective of anesthetic agent.

## 4 Discussion

This preliminary study showed that sevoflurane is associated with more frequent, severe PONV than propofol within the first 30 min after surgery. However, propofol and sevoflurane did not show any significant differences between the pre- and postoperative HRV dynamics. In the sevoflurane group, however, some frequency domain parameters [total power (Ln), LF, LF (Ln), and HF (Ln)] did not recover to the preoperative levels, and this phenomenon was markedly evident in the PONV group. Therefore, we infer a relationship between the decreased recovery of frequency-domain parameters and PONV.

Volatile anesthetics were the leading cause of early postoperative vomiting in the first 2 h postoperatively. The pro-emetic effect of volatile anesthetics was larger than other risk factors. The Kaplan–Meier curve revealed that the main difference between propofol and volatile anesthetic occurs within the first 2 h (3), which is consistent with our findings. In this study, the difference in the PONV profiles between propofol and sevoflurane were mainly seen immediately after surgery. The duration and severity of PONV and severity of nausea was higher in the sevoflurane group in the 30 min after surgery, and the intergroup difference in the PONV profile between the two agents disappeared thereafter. In this study, the overall incidence of PONV was not high (22.5%), which is attributable to the multimodal PONV preventive strategy and relatively short-duration anesthesia.

Parasympathetic activity is related to nausea and vomiting. Li et al. (11) reported that, compared to non-vagotomy patients, patients who underwent vagotomy developed lower PONV incidence. Morrow et al. (10) suggested that an increase in parasympathetic activity was related to the anticancer chemotherapy-associated nausea. Kanaya et al. (13) showed the differential effects of propofol and sevoflurane on the autonomic nervous system wherein propofol reduced cardiac parasympathetic tone, depending on the depth of anesthesia, whereas sevoflurane anesthesia had little or no effect on cardiac parasympathetic tone. Although we predicted differences in sympathetic/parasympathetic balance during recovery from propofol and sevoflurane anesthesia that would be related to PONV, the LF/HF ratio, which represents the sympathetic/parasympathetic balance, showed no significant differences between the sevoflurane and propofol groups, or between the PONV and no-PONV groups. This may be because the balance between sympathetic and parasympathetic activity does not constitute the main pathway among the various mechanisms of PONV in this study setting (2).

Interestingly, only diastolic blood pressure increased in the PONV group, while HR, SBP, and MAP remained stable. This may reflect localized vascular resistance changes rather than a generalized sympathetic surge, and partial baroreflex compensation might have contributed to maintaining overall hemodynamic stability. However, the concurrent reduction in HRV frequency components, including LF power, suggests that baroreflex sensitivity itself could have been impaired, indicating autonomic dysregulation rather than effective compensation. Given the small sample size, these interpretations should be made cautiously.

We had a reasonable suspicion that the recovery speed of the autonomic nervous system from anesthesia, rather than the LF/HF ratio, may be related to PONV. LF and HF power decrease with increasing depth of anesthesia (13, 17), and increased abruptly at the point wherein the patient became responsive to verbal commands (17). We measured HRV twice—preoperatively and in the PACU when the effects of anesthetic agents faded and PONV occurred. Between these two timepoints, significant changes in HRV were observed in the sevoflurane group, but not in the propofol groups. In the sevoflurane group, compared to the preoperative value, the total power (Ln), LF, LF(Ln), and HF(Ln) decreased postoperatively; this can be interpreted as slower recovery to the preoperative state, considering that HRV parameters decrease during anesthesia. This phenomenon was more pronounced in the secondary analysis where we compared the PONV and no-PONV groups. In the PONV group, as in the sevoflurane group, the frequency-domain parameters showed non-recovery to the preoperative state. The recovery speed differs depending on the anesthetic agent, and, even with the same agent, may have interindividual variations. Apfel et al. (3) suggested that immediate PONV in sevoflurane, compared to propofol, may be related to pharmacokinetics. In summary, the recovery speed of postoperative HRV parameters to preoperative levels may be related to PONV. However, this study did not generate conclusive evidence, which warrants further larger scale studies.

Compared to patients without PONV, patients with PONV had greater preoperative HF power, which suggests that the preoperatively increased parasympathetic activity is related to PONV (18). However, we found no difference in the preoperative HRV data between the PONV and no-PONV group. Several studies have shown that preoperative HRV data is related to perioperative outcomes, in addition to PONV, and these include preoperative high LF/HF ratio and hypotension after spinal anesthesia (19), preoperative high HF and bradycardia after spinal anesthesia (20), preoperative low total power and hypotension after induction of general anesthesia (21), etc. However, in their systematic review, Frandsen et al. (22) included numerous studies which showed that HRV lacks predictive power. The authors indicated that the potential role of preoperative HRV in conclusively predicting surgical outcomes is precluded by methodological heterogeneity, and warrants more high-quality research (22).

This study has some limitations. First, the small sample size of this preliminary study limits the generalizability of the results. Second, the short average anesthesia duration meant that the exposure time to each anesthetic agent was not sufficiently extended, contributing to the low overall incidence of PONV. Third, the relatively low incidence of PONV was attributable to the multimodal preventive strategy and the relatively short duration of anesthesia. Fourth, although blood pressure was monitored continuously throughout the perioperative period, only a single preoperative and postoperative value was used for analysis. This approach may not fully capture the dynamic nature of hemodynamic changes, and averaging multiple measurements could have provided more reliable data. Since the attending anesthesiologists were aware of group allocation, there is a potential for bias. However, as this was a preliminary study with no predetermined expectations, and because objective parameters like HRV were automatically recorded, we believe the impact of this limitation is minimal.

Our findings suggest a potential mechanistic link between anesthetic choice, autonomic nervous system modulation, and the incidence of PONV. Future studies should aim to confirm these associations through large-scale investigations. Furthermore, it would be valuable to conduct studies in settings with a higher incidence of PONV, such as longer surgeries or laparoscopic procedures.

While the choice of anesthetic agents influenced the extent of ANS recovery, the ANS dynamics linked to PONV could not be fully explained by anesthetic type alone. This finding suggests that the rate and extent of ANS recovery may be more directly related to the development of PONV. This perspective offers a potential new approach to PONV such as the modulation of the ANS could reduce the incidence of PONV. Further research is needed to elucidate the mechanisms underlying ANS recovery to preoperative levels and the factors that hinder this process.

To inform the sample size calculation for future studies, we performed an exploratory effect size estimation based on the data from our current study. Our findings suggested that changes in log-transformed low-frequency [ΔLF (log)] HRV values between the propofol and sevoflurane groups may be more meaningful than absolute values. Therefore, we focused our estimation on ΔLF [log], as this parameter appeared to be the most relevant indicator in our analysis. From our data, the mean ΔLF (log) was approximately −0.558 for the propofol group and −1.283 for the sevoflurane group, with pooled standard deviation of ∼1.40. This yielded an estimated Cohen’s d of 0.52, indicating a moderate effect size. Based on this effect size, we calculated that approximately 60 patients per group (total *N* = 120) would be required to detect a statistically significant difference in ΔLF (log) with 80% power at an alpha level of 0.05. This sample size increases to about 80 patients per group (total *N* = 160) for 90% power. Importantly, this sample size also adequately covers the incidence of postoperative nausea and vomiting (PONV). According to existing literature and our preliminary data, the expected incidence of PONV is approximately 10% in the propofol group and 35% in the sevoflurane group. For this anticipated difference, a sample size of approximately 47 patients per group is sufficient to achieve 80% power, and 63 patients per group for 90% power. Therefore, the sample size determined based on HRV biomarker analysis (ΔLF log) also provides sufficient power to detect clinically meaningful differences in PONV incidence. This ensures that both the physiological marker (HRV) and clinical outcome (PONV) are adequately powered within the same study design.

Sevoflurane use was associated with more frequent, severe PONV within the first 30 min after surgery than propofol use, despite no significant differences in HRV dynamics. Furthermore, in the sevoflurane and PONV group during the recovery period, the frequency-domain parameters were lower and failed to recover to preoperative levels, which may have been related to PONV. Despite the intriguing insights from this study, the data are insufficient for a definitive interpretation, and further large-sample studies are needed.

## Data Availability

The raw data supporting the conclusions of this article will be made available by the authors, without undue reservation.
